# Cytotoxic and apoptosis-inducing properties of a C_21_-steroidal glycoside isolated from the roots of *Cynanchum auriculatum*

**DOI:** 10.3892/ol.2013.1186

**Published:** 2013-02-07

**Authors:** LIANG-FEI YE, YI-QI WANG, BO YANG, RU-SONG ZHANG

**Affiliations:** Department of Pharmacy, Zhejiang Chinese Medical University, Hangzhou 310053, P.R. China

**Keywords:** *Cynanchum auriculatum*, steroidal glycoside, cytotoxic, apoptosis

## Abstract

The present study aimed to investigate the anti-cancer effect of a C_21_-steroidal glycoside (CG) isolated from the roots of *Cynanchum auriculatum*. CG was able to inhibit the growth of human cancer cells (SGC-7901 cells) in a concentration and time-dependent manner *in vitro*. SGC-7901 cells exposed to CG (10.8 and 21.6 *μ*M) exhibited typical morphological apoptosis characteristics, such as nuclear-chromatin condensation and apoptotic body formation. Flow cytometric analysis showed that after treatment with CG at 10.8 and 21.6 *μ*M for 24 h, the percentage of apoptotic cells increased to 30.4 and 43.2%, respectively, while the number of cells in the G_0_/G_1_, S and G_2_/M phases of the cell cycle decreased (P<0.05). Furthermore, treatment with CG at a concentration of 21.6 *μ*M for 24 h significantly increased the expression of caspase-3 and the activity of caspase-3 was increased ∼3-fold in SGC-7901 cells. These results suggest that CG is the active anticancer component of the total C_21_-glycosides of the roots of *Cynanchum auriculatum* which is able to inhibit the growth of cancer cells and induce cancer cell apoptosis through caspase-3-dependent pathways.

## Introduction

**Figure 1:**
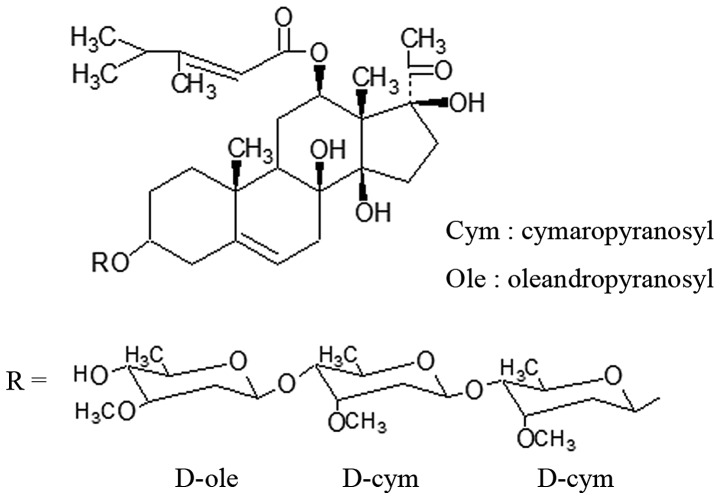
Chemical structure of CG.

The search for effective anticancer agents has been one of the most important areas in cancer control research. There are numerous natural plants used in clinical therapy for cancer in traditional Chinese medicine. The root of *Cynanchum auriculatum* Royle ex Wight, also known as Baishouwu, has been widely used in clinics since ancient times as an agent for anti-aging and prolonging life. Its major components, C_21_-steroidal glycosides (CG), are of considerable interest due to their bioactivities. Previous studies have revealed that the CGs isolated from Baishouwu were able to protect hepatocytes and neurons, as well as cells in the digestive system (1–3). Studies have also revealed the anticancer activity of these compounds (4–8). A number of CGs have been isolated from the roots of *Cynanchum auriculatum* Royle ex Wight. CG (Fig. 1) was one of the C_21_-steroidal glycosides obtained from Baishouwu and was first isolated by Warashina *et al* in 1995 (9). Few of the pharmacological functions of this compound have been reported. This compound attracted our attention due to its anticancer activity, which was more marked than that of the C_21_-steroidal glycosides previously reported (8). To further understand the potential anticancer properties of this compound, CG’s cytotoxic and apoptosis-inducing activities in several human cancer cells lines were studied.

## Materials and methods

### Cell lines

The human gastric cancer (SGC-7901), human colon cancer (HT-29) and human hepatoma (HEPG-2) cell lines were obtained from the Institute of Biochemistry and Cell Biology, Shanghai Institutes for Biological Sciences, Chinese Academy of Sciences (Shanghai, China). The cells were incubated at 37°C in humidified air containing 5% CO_2_.

### Concentration-dependent inhibitory effect assay

The SGC-7901, HT-29 and HEPG-2 cell viability was determined with an MTT assay. In brief, cells (3×10^3^) were seeded in 96-well microtiter plates with each well containing culture medium (100 *μ*l) supplemented with 10% FBS and incubated at 37°C overnight. The cells were then treated with CG (2.7, 5.4, 10.8, 21.6 and 43.2 *μ*M) or DMSO (0.1%, negative control). Following incubation for 48 h, 20 *μ*l of MTT solution (5 g/l) was added to each well. After 4 h of incubation at 37°C, the medium was discarded, DMSO (150 *μ*l) was added to each well and the optical density (OD) was measured at 570 nm using an ELISA Elx8000 plate reader (BioTek, Winooski, VT, USA). The cell viability was measured using the OD values. The rate of inhibition was calculated by the following equation: Viability inhibition = (ODc − ODt) / ODc × 100, where ODc is the optical density of the control group and ODt is the optical density of the drug-treated group. Based on the viability inhibition rate, the IC_50_ (concentration required to inhibit the cell viability by 50%) values were then calculated with NDST software (BioGuider Medicinal Technology, China).

### Time-dependent inhibitory effect assay

SGC-7901 cells were plated as described in the previous section. The cells were left to adhere overnight, then treated with CG (5.4–21.6 *μ*M) or 0.1% DMSO for 0, 24, 48 and 72 h. At the end of each treatment, the cell viability was determined using an MTT assay as described previously.

### Flow cytometry analysis

Propidium iodide (PI) staining was used to analyze cell apoptosis and the cell cycle. SGC-7901 cells (1×10^6^) were seeded in culture flasks prior to drug treatment. The cells were left to adhere overnight, then treated with CG (10.8 and 21.6 *μ*M) or DMSO (0.1%) for 24 h. At the end of the treatment, the cells were trypsinized and washed twice with ice-cold PBS, then fixed with ice-cold 70% ethanol in PBS at 4°C. The fixed cells were then centrifuged and washed with staining buffer. After washing, the pellets were treated with 100 *μ*l RNase A (1 g/l) for 30 min at 37°C. After the incubation, 900 *μ*l staining buffer and 20 *μ*l PI (1 g/l) were added to each sample and incubated in the dark for 30 min. The samples were then analyzed with a FACSCalibur flow cytometer (BD Biosciences, Franklin Lakes, NJ, USA).

### Morphological analysis

SGC-7901 cells (1×10^5^) were seeded in 6-well culture plates with each well containing medium (2 ml). The cells were left to adhere overnight, then treated with CG (10.8 and 21.6 *μ*M) or 0.1% DMSO for 24 h. Following drug treatment, the medium was discarded and the cells were washed twice with PBS. The cells were then observed with a light microscope (Olympus, Tokyo, Japan) or stained with acridine orange (5 *μ*l) for 10 min at room temperature in the dark and observed under a fluorescence microscope (Olympus).

### Determination of cleaved caspase-3 production

Cells (5×10^6^) in medium (20 ml) were transferred to each flask (75 cm^2^) prior to drug treatment. The cells were left to adhere overnight, then treated with CG (10.8 and 21.6 *μ*M) or DMSO (0.1%) for 24 h. At the end of the treatment, the cells were harvested and washed twice with ice-cold PBS, then lysed with lysis buffer (100 *μ*l). Proteins for the assay were obtained by collecting the supernatant and were used for the determination of cleaved caspase-3 by sodium dodecyl sulfate polyacrylamide gel electrophoresis (SDS-PAGE) and western blot analysis. The procedure was as follows: The protein concentration in the supernatant was determined using a BCA protein assay kit (KeyGen, Nanjing, China) with albumin as the standard. Equal amounts of proteins (60 *μ*g) from each group were loaded onto SDS-PAGE gels (12.5%) and electrophoresed to separate the proteins. The proteins from the gels were transferred onto nitrocellulose membranes. The membranes were blocked with non-fat milk (5%) in TBS (10 mM Tris and 100 mM NaCl) for 1 h and probed with primary antibodies (Santa Cruz Biotechnology, Santa Cruz, CA, USA) against cleaved caspase-3 and β-actin, followed by an appropriate horseradish peroxidase-conjugated secondary antibody (Zhongshan Bio-Tech Co., Ltd., Zhongshan, China) and ECL detection.

### Colorimetric caspase-3 activity assays

Caspase-3 activity was measured using a colorimetric assay kit (KeyGen) according to the the manufacturer’s instructions. In brief, the cells were plated and treated as described for the determination of cleaved caspase-3 production. At the end of the treatment, the cells were harvested, washed twice with ice-cold PBS and lysed for 60 min on ice in the lysis buffer provided in the kit. The proteins were collected by centrifuging at 10,000 × g for 1 min. The protein concentration in the supernatant was determined using a BCA protein assay kit (KeyGen) and samples were diluted to a concentration of 2 g/l using lysis buffer. Samples containing 50 *μ*g of proteins in lysis buffer (100 *μ*l) were added to the reaction buffer and caspase-3 substrates to measure the caspase-3 activity. The samples were incubated at 37°C for 4 h. The absorbance density was measured using a spectrophotometer (Amersham, Piscataway, NJ, USA) at 400 nm.

### Statistical analysis

The data are expressed as the mean ± SD. Values were analyzed with SPSS 16.0 software for Windows and the statistical significance of differences among the values was evaluated by one-way ANOVA. P<0.05 was considered to indicate a statistically significant difference.

## Results

### Concentration-dependent inhibitory effect of CG on three human cancer cell lines

**Figure 2:**
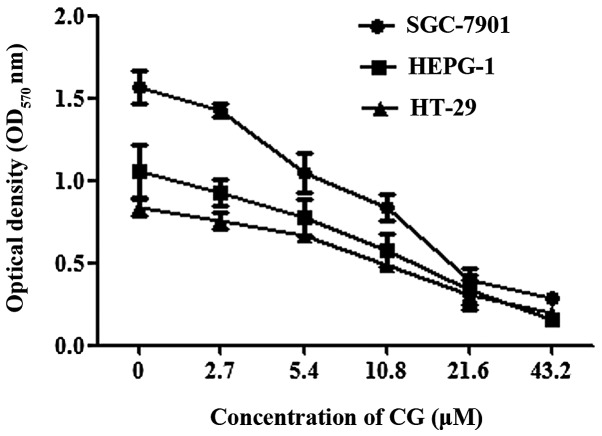
Concentration-dependent inhibitory effect of CG on three human cancer cell lines. The cells were treated with CG (0–43.2 *μ*M) for 48 h and MTT assays were used to analyze the cell viability. The data are presented as the mean ± SD, n=3.

The cytotoxicity of CG against three human cancer-cell lines; gastric cancer cell line (SGC-7901), colon cancer cell line (HT-29) and hepatoma cell line (HEPG-2), was determined. The cell viability was evaluated by measuring the mitochondrial metabolic activity of the cells using MTT assays. A concentration-dependent decrease in optical density at 570 nm (OD_570nm_) was observed following CG treatment in all three cell lines (Fig. 2). The cell viability inhibition rates were also calculated. Treatment with CG (2.7 43.2 *μ*M) for 48 h resulted in 11.9 to 85.3% decrease in cell viability in HEPG 2 cells, 10 to 75.9% decrease in HT29 cells and 9 to 81.4% decrease in SGC 7901 cells. The IC_50_ values of CG in the HEPG 2, HT 29 and SGC 7901 cell lines are 12.2, 16.4 and 12.6 *μ*M, respectively (Table I).

### Time-dependent inhibitory effect of CG on SGC-7901 cells

**Figure 3:**
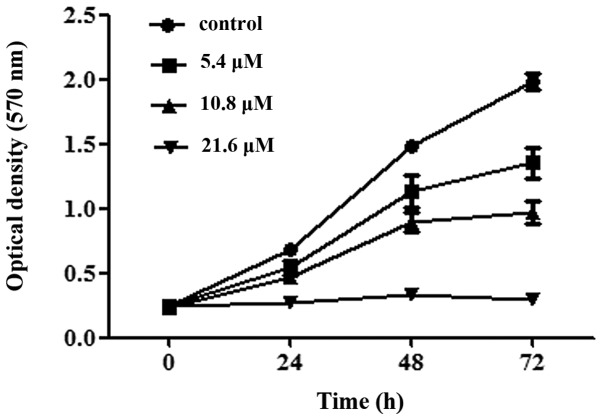
Time-dependent inhibitory effect of CG on SGC-7901 cells. The cells were treated with CG (0–21.6 *μ*M) for 0, 24, 48 and 72 h and MTT assays were used to analyze the cell viability. The data are presented as the mean ± SD, n=3.

To further understand the time-dependent inhibitory effect of CG, SGC-7901 cells were treated with CG (5.4–21.6 *μ*M) for 24, 48 and 72 h and an MTT assay was used to evaluate the cell viability. As shown in Fig. 3, the untreated SGC-7901 cells grew in an unrestrained manner and the OD_570 nm_ increased noticeably with the culture time. However, in the SGC-7901 cells treated with CG, the OD_570 nm_ increased slowly. At 21.6 *μ*M, the OD_570 nm_ did not increase with the culture time and the cell viability was inhibited by >50% compared with the control, even when the SGC-7901 cells were only treated for 24 h.

### Cell cycle distribution and apoptosis rate in SGC-7901 cells

**Figure 4:**
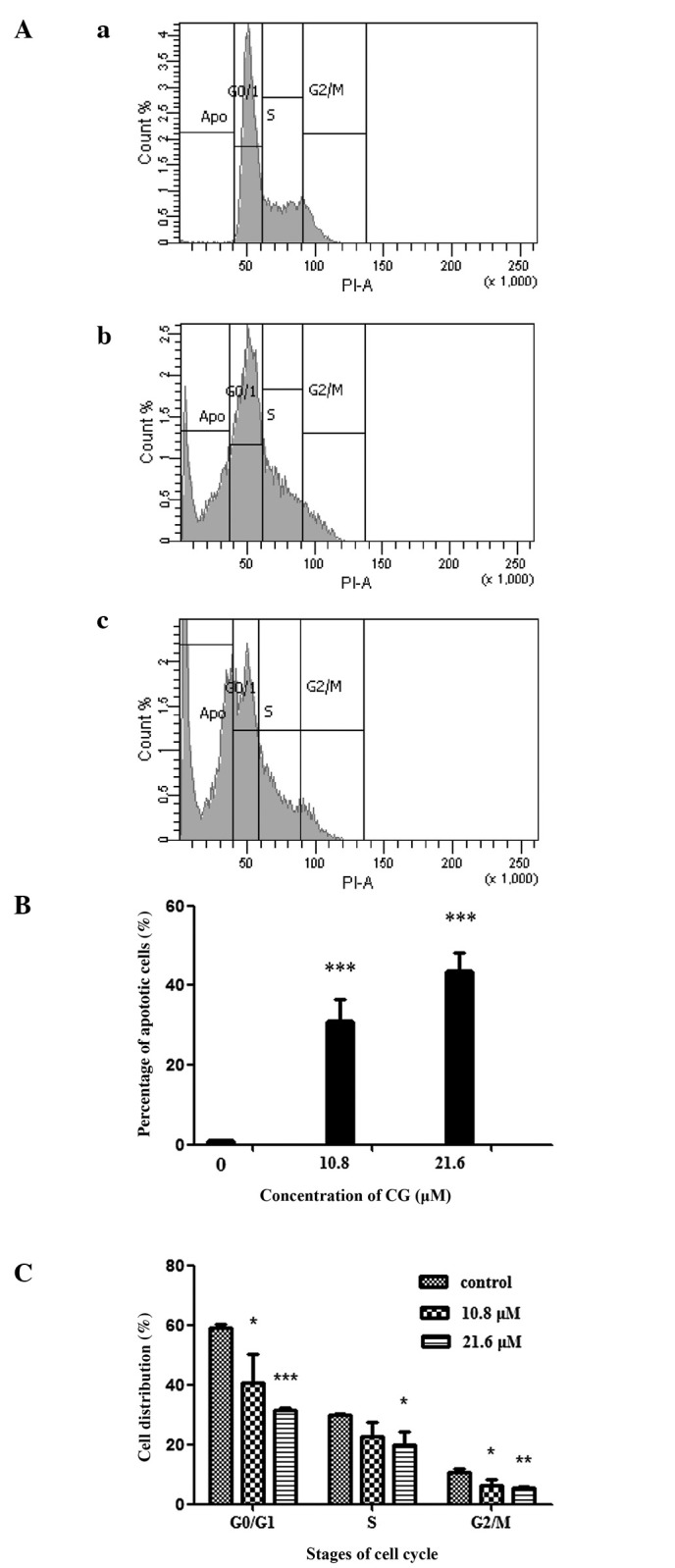
Effect of CG on apoptosis rate and cell cycle distribution in SGC-7901 cells. (A) Cell DNA content distribution in SGC-7901 cells with the treatment of various concentrations of CG. The cells were treated at drug concentrations of (a) 0, (b) 10.8 and (c) 21.6 *μ*M for 24 h and then stained with PI and analyzed by flow cytometry. (B) Apoptosis rate summarized from the cell DNA content distribution. (C) The percentage of cells distributed in each cell cycle summarized from the cell DNA content distribution. The data are presented as the mean ± SD, n=3. ^*^P<0.05, ^**^P<0.01, ^***^P<0.001, compared with the control. PI, propidium iodide.

In order to gain an improved understanding of the cell growth inhibition mechanism, the effects of CG on the cell cycle distribution and apoptosis rate of SGC-7901 cells were investigated. PI, a fluorescent dye, was used to stain the nuclear chromatin and flow cytometry was used to analyze cell apoptosis and the cell cycle according to the content of the cell chromatin. The total analyzed cells were separated into four groups; apoptosis, G_0_/G_1_, S and G_2_/M with regard to the distribution of DNA content. The gray area in Fig. 4A denotes the number of the cells in each group. As shown in Fig. 4, after the cells were treated with CG at 10.8 and 21.6 *μ*M for 24 h, the percentage of apoptotic cells increased to 30.4 and 43.2% respectively (P<0.001) while the cells in the G_0_/G_1_, S and G_2_/M phases decreased (P<0.05).

### Morphological changes in SGC-7901 cells

**Figure 5:**
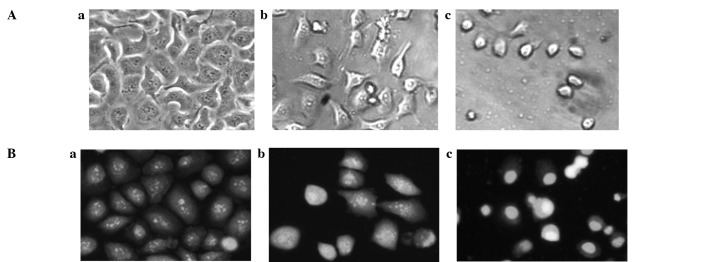
Morphological changes in SGC-7901 cells treated with CG. The cells were treated at drug concentrations of (a) 0, (b) 10.8 and (c) 21.6 *μ*M for 24 h and then (A) observed under a light microscope or (B) stained with acridine orange and observed with a fluorescence microscope. Magnification, ×400.

To confirm the apoptosis-inducing properties of CG, the morphological changes in SGC-7901 cells were studied. Under the light microscope, it was observed that the untreated SGC-7901 cells grew in an unrestricted manner with tight contact between neighboring cells. After being treated with CG (10.8 *μ*M) for 24 h, the gaps between the SGC-7901 cells became larger and a number of the cells shrank and became round in shape. When treated with CG at 21.6 *μ*M, the cell morphology changed significantly and the majority of the cells became round in shape (Fig. 5A). To observe the nuclear morphological changes, acridine orange, a fluorescent dye which binds to nuclear chromatin, was used. Under the fluorescence microscope, it was observed that the fluorescent dye was distributed evenly in the nuclei of SGC-7901 cells in the control group (Fig. 5Ba). When the cells were exposed to CG (10.8 and 21.6 *μ*M) for 24 h, clear apoptotic characteristics, such as chromatin condensation, nuclear fragmentation and apoptotic bodies, were observed in numerous cells. The morphological changes became more distinct when the concentration of CG was increased (Fig. 5Bb and c).

### Caspase-3 acivation in SGC-7901 cells

**Figure 6:**
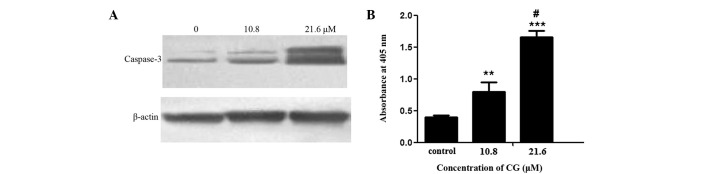
Effect of CG on caspase-3 activation in SGC-7901 cells. Cells were treated with CG (0–21.6 *μ*M) for 24 h and total cell lysates were prepared for (A) SDS-PAGE and western blot analysis or (B) enzymatic activity in the cell lysates was quantified by measuring chromophores obtained from the cleaved substrates. The data are presented as the mean ± SD, n=3. ^**^P<0.01, ^***^P<0.001, compared with the control. ^#^P<0.001 compared with the group treated with CG at 10.8 *μ*M. SDS-PAGE, sodium dodecyl sulfate polyacrylamide gel electrophoresis.

To further demonstrate the apoptosis-inducing effect of CG, the activation of protease caspase-3, which is activated in the classic apoptosis pathway and used as a marker of apoptosis induction (10), was studied. The results showed that CG (10.8 and 21.6 *μ*M) treatment for 24 h increased the production of cleaved caspase-3 in a concentration-dependent manner. As shown in Fig. 6A, the western blot analysis results revealed two bands at 19 and 17 kDa, which were the bands for cleaved caspase-3. The cleaved caspase-3 bands of the control were extremely faint. CG (10.8 and 21.6 *μ*M) treatment for 24 h increased the intensity of the two bands, particularly at 21.6 *μ*M. To further confirm the activation of caspase-3, a colorimetric assay kit (KeyGen) was used to examine the activity of caspase-3. The enzymatic activity of caspase-3 was quantified by measuring chromophores obtained from the cleaved substrates. As shown in Fig. 6B, after treatment with CG (10.8 and 21.6 *μ*M) for 24 h, the absorbance at 405 nm, denoting the caspase-3 activity, increased significantly (P<0.01). At 21.6 *μ*M, the activity of caspase-3 was increased ∼3-fold.

## Discussion

In our previous study, we isolated four new C_21_-steroidal glycosides from the roots of *Cynanchum auriculatum* and reported the anticancer activity of auriculoside A (AA) which was the most active of the four (8,11,12). In the present study, CG, a C_21_-steroidal glycoside first isolated by Warashina *et al* in 1995, demonstrated greater cytotoxic effects than AA. The IC_50_ values of CG, which ranged between 12.2 and 16.4 *μ*M, were lower than those of AA, which ranged between 23.2 and 36.7 *μ*M (8). Furthermore, the IC_50_ values of CG were lower than the C_21_-steroidal glycosides reported by other studies. For example, the IC_50_ value of caudatin, a C_21_-steroidal aglycone from the roots of *Cynanchum auriculatum* was reported to be 84.51 *μ*M at 48 h in HEPG-2 (13). The great difference in the inhibitory effects of these C_21_-steroidal glycosides in similar cancer cells suggests that there may be certain rules governing the association between the structure and activity of this type of compound.

Apoptosis induction is a key event that is the target of numerous chemopreventive agents (13,14). C_21_-steroidal glycosides also show a clear apoptosis-inducing effect (6,8,13). Accordingly, the apoptosis-inducing properties of CG were investigated in the present study. The results showed that CG induced cancer cell apoptosis at a lower concentration than a number of other C_21_-steroidal glycosides (6,8,13). As analyzed by flow cytometry, 21.6 *μ*M CG treatment for only 24 h induces apoptosis in >40% cells and this is consistent with its strong cytotoxicity in cancer cells *in vitro*. Morphological changes are clear evidence of apoptosis. Apoptotic cells are characterized by distinct morphological features, including cell shrinkage and loss of contact with neighboring cells, chromatin condensation, nuclear fragmentation and apoptotic body formation. To demonstrate the apoptosis-inducing effect of CG, the cell outline and nucleus were observed under light and fluorescence microscopes. It was revealed that SGC-7901 cells treated with CG exhibited typical apoptotic morphological features. Usually in apoptosis, caspase-3 is activated and this is often used as a marker of apoptosis induction (15). Activated caspase-3 is cleaved into two segments (cleaved caspase-3) with molecular weights of 17 and 19 kDa. Cleaved caspase-3 is able to activate deoxyribonuclease, leading to DNA fragmentation and apoptotic cell death (16). In the present study, the production of cleaved caspase-3 and the activity of caspase-3 were also investigated to assess caspase-3 activation. The results showed that the production of the two segments of cleaved caspase-3 and the activity of caspase-3 increased following CG treatment for 24 h particularly at 21.6 *μ*M. Thus the apoptosis-inducing effect of CG was further demonstrated.

In conclusion, the present study demonstrated the cytotoxic and apoptosis-inducing properties of CG and showed that CG is a potent anticancer C_21_-steroidal glycoside from the roots of *Cynanchum auriculatum*. This study also emphasized the importance of comparing the pharmacological properties between the various C_21_-steroidal glycosides. In future research, emphasis should be placed on identifying the rules governing the association between the structure and activity of this type of compound.

## Figures and Tables

**Table I:** IC_50_ values of CG against three human cancer cell lines (mean ± SD, n=3).

Cancer cell line	Cell type	IC_50_ (*μ*M)
HEPG-2	Liver	12.2±0.6
HT-29	Colon	16.4±2.1
SGC-7901	Stomach	12.6±0.5
